# Applying a Commercialization-Readiness Framework to Optimize Value for Achieving Sustainability of an Electronic Health Data Research Network and Its Data Capabilities: The SAFTINet Experience

**DOI:** 10.5334/egems.295

**Published:** 2019-08-29

**Authors:** Elaine H. Morrato, Mika K. Hamer, Marion Sills, Bethany Kwan, Lisa M. Schilling

**Affiliations:** 1 Department of Health Systems, Management and Policy, Colorado School of Public Health, University of Colorado Anschutz Medical Campus, US; 2 Adult and Child Consortium for Health Outcomes Research and Delivery Science (ACCORDS), University of Colorado Anschutz Medical Campus, US; 3 Departments of Pediatrics and Emergency Medicine, School of Medicine, University of Colorado Anschutz Medical Campus, US; 4 Department of Family Medicine, School of Medicine, University of Colorado Anschutz Medical Campus, US; 5 Division of Internal Medicine, School of Medicine, University of Colorado Anschutz Medical Campus, US

**Keywords:** electronic health data networks, FQHC, CER, PCOR, dissemination, implementation

## Abstract

**Context:**

Sustaining electronic health data networks and maximizing return on federal investment in their development is essential for achieving national data insight goals for transforming health care. However, crossing the business model chasm from grant funding to self-sustaining viability is challenging.

**Case description:**

This paper presents lessons learned in seeking the sustainability of the Scalable Architecture for Federated Translational Inquiries Network (SAFTINet), and electronic health data network involving over 50 primary care practices in three states. SAFTINet was developed with funding from the Agency for Healthcare Research and Quality to create a multi-state network for comparative effectiveness research (CER) involving safety-net patients.

**Methods:**

Three analyses were performed: (1) a product gap analysis of alternative data sources; (2) a Strengths-Weaknesses-Opportunities-Threat (SWOT) analysis of SAFTINet in the context of competing alternatives; and (3) a customer discovery process involving approximately 150 SAFTINet stakeholders to identify SAFTINet’s sustaining value proposition for health services researchers, clinical data partners, and policy makers.

**Findings:**

The results of this business model analysis informed SAFTINet’s sustainability strategy. The fundamental high-level product needs were similar between the three primary customer segments: credible data, efficient and easy to use, and relevance to their daily work or ‘jobs to be done’. However, how these benefits needed to be minimally demonstrated varied by customer such that different supporting evidence was required.

**Major Themes:**

The SAFTINet experience illustrates that commercialization-readiness and business model methods can be used to identify multi-sided value propositions for sustaining electronic health data networks and their data capabilities as drivers of health care transformation.

## Background

The number of electronic health data networks for policy-informing health services research and patient-centered outcomes research (PCOR) has proliferated significantly in the United States over the past two decades [1]. One of the oldest electronic research data networks is the Health Care Systems Research Network, formerly known as the HMO Research Network. This network, a national consortium of 20 research departments within health care delivery systems, began coordinating federally-funded scientific networks and studies in 1994 [2]. In 2007, the DARTNet Institute, formerly the DARTNet Collaborative, was formed in partnership with the American Academy of Family Physicians National Research Network to support practice-based research networks and serve as an umbrella organization for networks seeking to use electronic health data for comparative effectiveness research, quality, safety, and to support the learning health system [3]. The American Recovery and Reinvestment Act of 2009 further accelerated the development of other research data networks by investing one-third of its $1.1 billion funding to improve data capacity [4,5]. The Patient Centered Outcomes Research Institute (PCORI) continued this investment awarding over $250 million in 2014 to support ongoing development, expansion, and use of PCORnet, a National Patient-Centered Clinical Research Network. PCORNet is comprised of 33 individual health data networks, which include 13 clinical data research networks and 20 people-powered research networks, and two health plan research networks [6].

Sustaining electronic health data networks and maximizing return on federal investment in their development is essential for achieving national data insight goals for transforming health care. eGEMS published a series of articles on lessons learned in efforts to sustain the effective use of clinical research data infrastructures and their electronic health data networks and maximize the return on investment in these networks. Three prominent themes emerged: the importance of data network maturity, commercial viability considerations, and stakeholder support [7]. At the time, the editors noted that additional case studies of commercialization efforts were needed to demonstrate the application of this sustainability strategy.

Launching and financially sustaining a new product or enterprise – like a new electronic health data network – is challenging. Real-world commercialization experience suggests that three-quarters of tech startups fail primarily due to a lack of customers not due to poor technology execution [8]. Osterwalder, Pigneur and colleagues write that to commercially succeed, new start-ups must satisfy three criteria necessary for sustainable customer acquisition: Problem-Solution fit, Product-Market fit, and Business Model fit [9]. Problem-Solution fit means that the technology is designed to address important unmet needs for the customer. The product’s value proposition states the important differentiating benefits customers can expect versus what is currently available from competing alternatives, including the status quo. The value proposition serves as a business hypothesis to be validated through stakeholder engagement and customer feedback. Start-up development teams that focus on clearly identifying their target customer and value proposition perform twice as well in business pitch competitions to secure funding than teams that do not [10]. Product-Market fit occurs when there is evidence that the product or service actually delivers the hypothesized value in the market and there is growing customer demand [9]. Business Model fit occurs when the value proposition can also be embedded in a financially sustainable and scalable business model and move beyond being a demonstration project only.

This paper seeks to describe the case experience of using a business model framing to achieve sustainability of one electronic health data network, the Scalable Architecture for Federated Translational Inquiries Network (SAFTINet). We describe the systematic application of customer discovery and operational research to help improve network services and data use value for our target stakeholder customers.

## Case Description

SAFTINet was developed with funding from the Agency for Healthcare Research and Quality to create a multi-state network for comparative effectiveness research (CER) with a focus on vulnerable populations. Health care disparities, the care of underserved, minority, and rural populations, and conditions more common in socioeconomically disadvantaged populations were well represented in the Institute of Medicine’s (IOM) top 100 high-priority CER topics [11]. A core benefit of the secondary use of electronic health data is the ability to study the care of patients in day-to-day practice, where conditions that impact variability in care and health outcomes are taken into account [12]. CER has particular appeal as a method to assist in gaining a better understanding of many health care disparities and conditions that tend to be more prevalent or more severe in safety-net populations. CER that addresses minority, underserved, and rural populations is especially valuable due to their historically limited representation in clinical research, well-documented health care disparities, and the differences between documented clinical trial efficacy and real world effectiveness in these populations [13].

**Figure 1:**
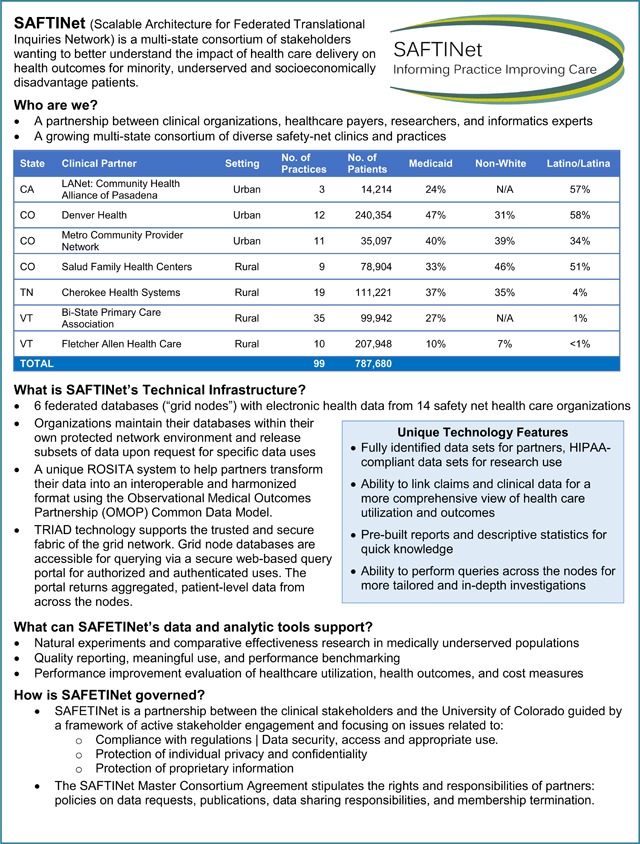
Product description.

Figure 1 is a fact sheet that describes ***SAFTINet’s Product Features*** at the time the sustainability analysis was conducted. SAFTINet had four partnering safety-net clinical practice organizations, with 54 participating primary care practices (federally qualified health centers [FQHCs] or FQHC look-alikes) in Colorado, Vermont, and Tennessee that were all adopters of electronic health record (EHR) systems. These urban and rural practices include family medicine, internal medicine, pediatrics, and behavioral health clinicians who care for approximately 260,000 unique safety-net patients per year.

SAFTINet is a distributed data network [14], with locally-controlled databases of administrative, clinical, Medicaid claims and patient-reported outcome (PRO) data which can be used for a broad range of research, quality improvement, and care delivery purposes [15]. There is a formal governance structure and organizations release approved datasets upon request to researchers.

SAFTINet’s data infrastructure, which includes robust conventions to the OMOP common data model [16], is capable of supporting a variety of use case applications of potential value to various data users (or in other words, ‘customer segments’), including use for:

- Observational health services research, including comparative effectiveness, cost-effectiveness and patient-centered outcomes research
- Pragmatic comparative effectiveness clinical trials
- Quality reporting, meaningful use, and performance benchmarking
- Multi-institution collaborative investigation to identify best practices
- Data quality assessment and validation

## Methods

Three analyses commonly used to assess market need and fit were performed to inform SAFTINet’s sustainability planning:

1. *Product Gap* analysis for understanding the competitive environment, in this case electronic health data alternatives which could be used for conducting health services research involving vulnerable populations;
2. *Strengths-Weaknesses-Opportunities-Threat* (SWOT) analysis for examining SAFTINet’s strategic market fit within the environment of competing health data alternatives; and
3. *Customer Discovery* for identifying SAFTINet’s strongest value proposition for its target customers and partners including health services researchers, safety-net clinical data partners, and policy makers.

### Product Gap Analysis

In business planning, the product gap (also called a segment or positioning gap) is the part of the market that is not currently being optimally served. The gap represents a competitive opportunity for new market entrants. We considered alternative products to be other electronic health networks and data sources that provided access to clinical data and/or claims data available at the time of our business analysis (2013–2015). Electronic health data products were categorized based on their characterizing patient populations and type of electronic health data. Safety-net populations include uninsured Americans and those who are Medicaid beneficiaries; therefore, patient populations were defined by insurance coverage. The types of electronic health research data were sub-divided into national survey and panel data, administrative claims data, EHR data, and linked administrative and EHR data.

### Strengths-Weaknesses-Opportunities-Threat (SWOT) Analysis

A SWOT matrix is a strategic exercise and planning tool for evaluating a project venture [17]. The degree to which the internal environment of an organization (its strengths and weaknesses) match the external market environment (opportunities and threats) contributes to Business Model fit. Gaps in fit highlight focus areas for product and business development. The SAFTINet research team developed and refined its SWOT analysis based on internal knowledge from engagement with its development partners and on external feedback from researchers and safety-net clinicians not directly involved in the project.

### Customer Discovery

The specific aim of the customer discovery process is to articulate and validate a product’s unique value proposition relative to the alternative options customers can choose instead [9]. Value can be derived by translating product features into benefits via two ways: as “gain creators” in which the product helps the customer achieve outcomes important to their job and as “pain relievers” in which the product helps the customer avoid bad outcomes, risks and obstacles. Strong value propositions help customers do their jobs better. In this context, jobs are not necessarily just functional tasks (e.g. improving health care quality, reducing costs) but can also include social goals (e.g., improving reputational status) and emotional goals (e.g., achieving peace of mind or job security). Through customer discovery, researchers listen and uncover the most important jobs their customers are trying to accomplish and their most critical pains and gains in doing those jobs. By listening to customers, researchers can identify the strongest value proposition for their product and where they may need to pivot their product offering in order to be successful.

Customer discovery is a form of stakeholder engagement. Practice-based research networks, like SAFTINet, are built upon a foundation of mutual engagement and research value creation [18,19]. For the SAFTINet project, a variety of stakeholder engagement methods was undertaken [20] and provided a forum to listen and validate SAFTINet’s value with its customers. In total, customer discovery interviews and stakeholder engagement occurred with approximately 150 SAFTINet stakeholders and potential users.

Methods are stakeholder engagement were:

1. Annual Partner Face-to-Face Convocations
  SAFTINet partners convened for day and a half long in-person meetings on an annual basis over several years during the technology development phase. The purpose of the convocations was to discuss the state of the network and obtain feedback on opportunities to improve the data infrastructure. Each clinical partner sent several representatives to these convocations, including organizational leadership (e.g., medical director), the SAFTINet site coordinator, and a technical team member (e.g., analyst). At the partner convocation in February 2014, an expanded set of stakeholders were invited to participate using the *7 P’s of Stakeholder Engagement* as our taxonomy for identifying and engaging patient, provider, payer, policymaker, provider, principal investigators, and product manufacturer stakeholders [21].
  A skilled external facilitator led the group in drafting SAFTINet value propositions, clarifying the network mission statement, and exploring opportunities for continued development of the network infrastructure using the ToP® Focused Conversation method [22,23]. The focused conversation method was developed by the Institute of Cultural Affairs in Ontario and has been applied to a variety of organizational settings to frame public input sessions, to capture participant feedback, and to apply new information to an existing plan or activity. It provides a roadmap for designing a discussion guide that elicits meaningful dialogue and ideas through structured group participation and promotes shared understanding. Conversation was focused in four parts: (1) objective questioning (What data do we have? What questions do we want to answer?); (2) reflection (Do these data needs bring to mind other similar initiatives or collaborations from which we can learn?); (3) interpretation (Why are these data and questions important to us?); and (4) decision making (Where should SAFTINet focus on first to optimize value?).
2. Regular clinical partner web conferences
  SAFTINet partner representatives and central project personnel meet regularly via web conference (frequency ranging from twice monthly to quarterly depending on network activity) as previously described (20). Web conferences were used to gather partner input on ways in which SAFTInet data could provide value to the data-providing partners in clinical practice; this input was used to corroborate and refine value propositions hypothesized from the other engagement activities.
3. Product concept and customer discovery interviews
  Supplemental in-depth qualitative interviews were conducted with 14 individuals representing the three key SAFTInet target customers: CER researchers, safety-net clinicians, and policy makers and informants. These individuals were naïve to SAFTINet (i.e., were not members of network partners and had not participated in previous SAFTINet engagement activities or used the network infrastructure). Discussion topics included an exploration of unmet electronic health data needs, feedback on proposed value propositions for SAFTINet, and exploration of the claim support needed as evidence to support the value proposition. A card-sorting technique was used for participants to rank order which needs and evidence was most important to them and for the interviewer to elicit their rationale for the rankings. Pricing sensitivity was also explored.
  The interviews were digitally recorded and transcribed. The credibility and authenticity of the qualitative customer discovery learning was enhanced by using several recommended strategies: (1) semi-structured interview guides administered by an experienced facilitator; (2) well-defined purposeful sampling of stakeholders; and (3) qualitative content analysis methods using systematic coding with ATLAS.ti software [24,25].

## Results

**Figure 2:**
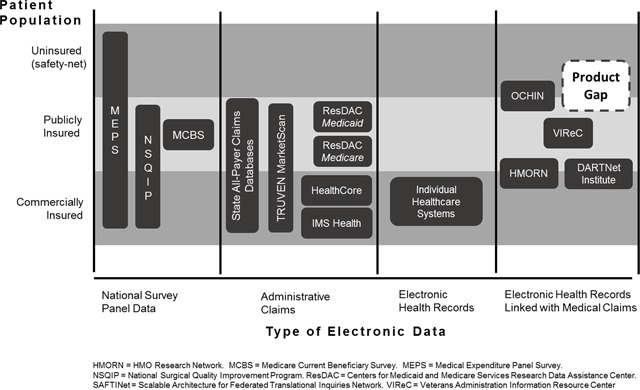
Product gap analysis for electronic health care research data in the United States at the time of SAFTINet’s sustainability evaluation.

Figure 2 shows our ***Product Gap Analysis*** for electronic health data sources in the United States available to study safety-net populations at the time SAFTINet was being developed. They represent the competitive alternatives to SAFTINet that health services researchers, who conduct comparative effectiveness research, could select to purchase or use. During 2013–2015, when the analysis was performed, there were limited publicly-available electronic health record data options for health services researchers interested in comparing care in safety-net clinical populations.

The health services researchers interviewed mentioned several public data sources they routinely used, but that they found lacking for comparative effectiveness research in uninsured safety-net populations. For example, health service researchers routinely purchase and use national survey panel data. The Medical Expenditure Panel Survey (MEPS) is one type that includes uninsured and under-insured Americans. MEPS is a set of large-scale surveys of families and individuals, their medical providers, and employers across the United States; MEPS represents the most complete source of data on the cost and use of health care and health insurance coverage [26]. However, MEPS has a two-year lag in data availability and, while large, it is under-powered for product- or disease-specific comparative effectiveness research questions, especially within vulnerable populations. Moreover, it is aggregated at the national level and therefore not useful for clinic-level benchmarking and performance improvement.

Administrative claims data were available for publicly and commercially-insured populations, but not for safety-net populations. For example, Medicaid Adanalytic data extracts are available for research through the Research Data Assistance Center (ResDAC) national repository, but there is a multi-year lag in data availability for researchers unless they have a direct relationship and data use agreement with individual states. Moreover, Medicaid data are administrative claims data used for billing; information on clinical measures important for safety-net populations and necessary for many research questions (e.g., uncontrolled hypertension, smoking status, asthma control) is absent.

Electronic health record data were available primarily within commercially insured health systems who could afford purchasing electronic health record software. Similarly, patient-level electronic health data linking claims and electronic health record data existed, but primarily for commercially insured patients (e.g., HMORN) or for narrowly defined groups (e.g., VIReC and veterans).

The product gap analysis showed that electronic health data involving safety-net health care clinics was a meaningful market gap. At the time, OCHIN was identified as the only alternative research data provider in the safety-net space with the capability of providing linked EHR and administrative claims data [27,28]. OCHIN is a non-profit health care information network based in Oregon. It is not affiliated with an academic health center but instead is comprised almost exclusively of federally qualified health centers and rural health centers. Its mission is to encourage research with potential to directly benefit safety net patients, to develop and improve OCHIN’s data resources for research purposes, to partner with researchers, and to translate research findings into practice [29]. OCHIN is now part of a PCORI Clinical Data Research Network (ADVANCE). Although a sizable network (93 organizations serving upwards of 1.4 million patients), OCHIN’s reach was limited to 18 states.

**Figure 3:**
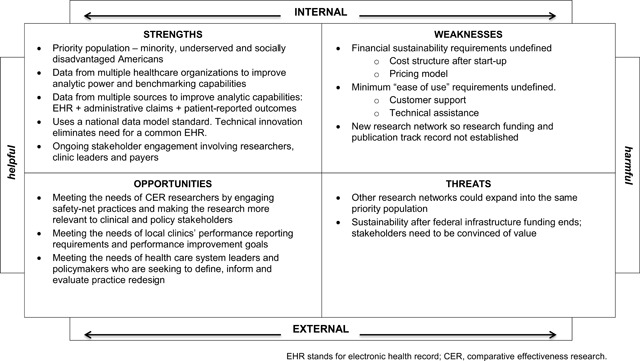
Strength-Weaknesses-Opportunity-Threats (SWOT) analysis for SAFTINet.

Figure 3 shows the ***SWOT Analysis*** for SAFTINet. At the time the analysis was conducted, several strengths emerged. They included SAFTINet’s use of a national data model standard that was designed to standardize both EHR and claims data, its progress with acquiring and linking Medicaid claims data with the EHR data, and its ongoing stakeholder engagement involving clinic leaders, payers and researchers. An unmet need identified for safety-net practices was the ability to generate reports and perform data analytics using their local data to inform quality and performance improvement initiatives. While advanced performance reporting and value-based care analytics were being rapidly developed and marketed by outside EHR vendors, these big-data analytic advances were still out of reach for many groups [30], particularly resource-limited safety-net organizations.

SAFTINet’s major strategic weakness was the absence of a sustaining revenue model beyond the initial infrastructure development funding from federal sources, which was the reason this work was undertaken. Given the funding source, CER researchers were the original target customer. However, to achieve sustainability the data-sharing clinical partners and policy makers (who make funding decisions affecting safety-net health care delivery) were also identified as critical target customers for whom value creation could also be demonstrated. The next step was to define the value proposition from each customer perspective.

Based on the product gap and SWOT analysis, we hypothesized SAFTINet had several features that could differentiate it from the other electronic health data sources and provide value. First, it is a safety-net focused data network. Second, it has focused on combining clinical and Medicaid claims data at the patient-level for greater data breadth and data granularity. Third, it was one of the first clinical research networks to use an internationally recognized common data model, the Observational Medical Outcomes Partnership (OMOP) Common Data Model and vocabulary [16] and EHR-agnostic method for aggregating and coding the data. It is a practice-based research network and learning community of safety-net stakeholders who are committed to addressing research questions relevant for the populations they serve [20].

Table 1 summarizes the ***Value Proposition insights from the Customer Discovery process***. In the end, the fundamental high-level product needs were the same across the three different customer segments (or stakeholder types) – credible data, efficient and easy to use, and work relevance. However, how these benefits needed to be demonstrated varied by stakeholder. The respondents indicated that data use cases would be essential to demonstrate the incremental value of SAFTINet versus current practice and competing alternative data sources.

- **Credible data** for a researcher meant it held up to the scrutiny of academic peer review; for a clinical provider it meant it was consistent with historical clinical reporting within the center; for a policymaker it meant it held up to public scrutiny and there was trust in the data organization itself.
- **Efficient and easy to use** for a researcher meant data access within one month of a request and a well-documented data dictionary; standardized performance reports provided monthly for clinical providers; and ready-to-use key metrics for policy makers to use on-the-spot in discussions with constituents. Importantly, customer service and technical assistance were identified as minimal viable product characteristics for clinical and policymaker users when they defined easy to use. The timeliness of data acquisition was key for all stakeholders, any governance or policies that slowed access to data or to the ability to publish research results (especially, for those with shorter funding cycles) was viewed as a negative. This means that SAFTINet viability is dependent upon an efficient data governance and use agreement process.
- **Work relevance** for researchers meant electronic health data that could allow meaningful scientific contributions as measured by peer-reviewed publication. Demonstration of data validity was essential to this need. Researchers who sought to conduct pragmatic clinical trials or other prospective interventional research saw the value of SAFTINet’s stakeholder-engaged network as a means for facilitating that data collection (e.g. patient reported outcomes).
  For clinical data partners, work relevance meant making performance reporting requirements more efficient. There was interest in linking SAFTINet with other population-based data, like social determinants of health, for a more granular understanding of factors affecting the health of their safety-net patients.
  For policy makers, work relevance meant the ability to conduct more targeted effectiveness analyses using cost data from the claims data linked with clinically important patient characteristics available in the EHR data). For example, SAFTINet could provide the ability to examine the impact of a program to improve blood pressure control among patients whose blood pressure is clinically elevated (added value) vs. among patients for whom only their prescription medication status is known (current situation).

**Table 1:** Value proposition.

Value proposition		Customer Segment		

		Health Services Researcher	Clinical Providers and Health Systems	Policymakers and Informers

SAFTINet is the linked EHR-claims data source for safety-net populations…		… that provides easy-to-access, credible data for comparative effectiveness research.	… that provides credible and actionable reporting for performance benchmarking.	… that provides credible and timely information for policy and program planning and evaluation.

**Attribute: Valid and credible data source**	**What I need …**	Scientifically validated data … … for grant proposals and publishing in top tier journals (my standard: academic peer review)	Data output consistent with what I know about my clinic population… … for quality improvement and performance reporting (my standard: historic clinical reporting)	Convincing evidence … … for policy and program decisions (my standard: publicly defendable)
	**Illustrative quotes**	*We know what’s in there … how the sample was drawn… people trust the information*	*Show me that what I get out is the same as what I am putting into it*.	*Currently no data are available to validate the state’s FQHC scorecards*.
	**What evidence will convince me …**	Data provenance validation Federally-funded research Scientific publications	Clinical corroboration	Credibility and track-record of the people processing the data
**Attribute: Efficient and easy to use**	**What I need …**	Quick access to cleaned data … … so I can complete my research analysis on time. (my standard: 1 month)	Quick access to reports … … so I can monitor and track performance improvement goals. (my standard: monthly)	Simple, quick summary numbers … … so I can discuss implications with constituents as I sit with them. (my standard: today)
	**Illustrative quotes**	*Most of my studies tend to be pretty short. 12–24 months… if you are dealing with datasets that are not prepared you’re realistically in 6–12 months of cleaning [and] data preparation, then I’d rather just start with a clean dataset …*	*We are a government institution.. before we can advocate for improvement … we have to show that we’re doing what is mandated. And the quicker and the more efficiently we can do that, the more quickly and efficiently we can get on to [improvement] that actually matters*.	*Probably the most driving force behind [our data users], and we hear that all the time*.
	**What evidence will convince me …**	Data dictionary Point-and-click summary data functionality (for grant proposals) Ready-to-use IRB/DUA language	Ready-to-go report templates Track-record statistics on timeliness of standard reporting Custom reports and support services	Point-and-click summary data functionality (for conversations with constituents) Custom queries and support services
**Attribute: Relevance and usefulness to my daily work and priorities…**	**What I need …**	The ability to make meaningful and respected scientific contributions.	The ability to do better clinical improvement.	The ability to target my policies and measure their financial impact.
	**Illustrative quotes**	*It feels to me like the communities that have a powerful situation like [linked EHR and claims data], like Harvard Pilgrim … Kaiser, really important stuff comes out of there*.	*The more data that we have on a variety of topics, the better we can make decisions on how we want to treat in the future and also what kind of different [clinical improvement] options we want to open up*.	*It’s all about cost right now. I mean we have a triple aim, and I always put cost third, because I don’t want to forget the other two things, but the reality is that we are in the middle*
		*… we definitely are entering the world of pragmatic trials, but I still also do a lot of secondary analysis, so having that diversity of research being supported is important*.	*[we need to know] this is how we’re doing, and I think it can help [to compare to similar sized practices with similar resources], … we’re a small practice and we don’t really have a versus [benchmark] ___.”*	*of a crisis of cost, and if we don’t get that under control we can’t do any of the other stuff*.
	**What evidence will convince me …**	Examples of cited research Variety of research methods supported	Examples of performance improvement initiatives	Examples of policy evaluation and cost effectiveness

The customer discovery process also explored varying price sensitivities for each customer. A health services researcher requiring data for secondary analyses for a small-grant (<$250,000) expected to pay less than $25,000 for a dataset (ideally $10,000) based on their current data acquisition costs. Acceptable costs increased to $50–75,000 for researchers with RO1-level research funding and more substantial data needs, like patient-reported outcomes data, which required new data collection. On the other hand, safety-net practices were more familiar with subscription fee models for clinical informatics support. Therefore, given their limited budgets, they expected to pay no more than $2000–5,000 annually to participate in data-sharing and receive quarterly quality performance reports. If one assumes a $300,000 a year operating cost to maintain SAFTINet’s data infrastructure and service and to support data partner participation, then the SAFTINet enterprise would need an estimated 12 data requests per year if targeting small grant researchers, 4–6 large grant researchers, or 60+ practice subscriptions to break even financially.

## Discussion

During the three-years of federal data infrastructure development funding from AHRQ, great technical progress occurred in standing up a new distributed safety-net data research network, including the creation of governance policies, clinical and claims data acquisition procedures, and novel informatics solutions for achieving data harmonization. However, three years was insufficient to also demonstrate value to all of SAFTINet’s stakeholders in order to self-sustain the data network. Recognizing that federal funding was not available to maintain the CER data networks just developed, AHRQ instead provided competitive supplemental funding to help networks conduct stakeholder engagement and have additional development time for optimizing stakeholder value.

Successful entrepreneurs learn to continuously adapt and pivot, i.e., change product strategies to better meet customer and market demands [31]. Systematically applying a business model framework to investigate the needs of SAFTINet’s customers and how to optimize network value for them was vital in changing SAFTINet’s strategies for achieving sustainability. First, applying a commercialization lens confirmed that a business model would not be viable if it did not provide tangible value to both health services researchers (data users) and clinical partners (data contributors). Second, the investigation revealed specific minimum viable product characteristics and the associated claim support (or proof-of-concept) evidence required to demonstrate value to different customers of the data. Lastly, the process provided insights on the number of customers (whether it be in the form of data requests or license agreements) necessary for SAFTINet to financially break even. This last analysis demonstrated the significant leap, and the resulting business enterprise investment required, for federally-funded academic enterprises to immediately acquire new paying customers once federal start-up development funding ends.

The findings were used to explore common ***Business Models*** that could be applied to sustain an electronic health data network [32], as shown in Table 2. SAFTINet’s current sustainability approach is a mixed model – Free and Open. It is based on sustaining and underwriting the partnerships with SAFTINet’s clinical data partners (the Free business model) by facilitating participation in meaningful research activities and collaborating on building tools to assist with high-value data use such as performance reporting. Unlike initial infrastructure funding which provided support for activities that were novel, like sustaining the collection of patient-reported outcomes for research purpose, partner responsibilities were reduced to those that are essential, such as data transformation, and not maintenance of technology that supports automated distribution of federated queries. Datasets are now easily transferred via sFTP. Collaborations with OHDSI, PCORNet- specifically pSCANNER, and the DARTNet Institute are ongoing to maintain value for health services researchers (the Open business model). This represents the strategic choice to diversify partnerships, i.e., be part of a network of networks, for quicker and broader access to potential research customers than a go-solo business model. Once high-value data use tools are developed and demonstrated within SAFTINet, the network plans to implement a hybrid subscription/research data use model with different pricing levels for different customer segments.

**Table 2:** Business models.

	Unbundling Business Models	The Long Tail	Multi-Sided Platforms	FREE as a Business Model	Open Business Model

**Description**	The business is separated into complementary models dealing with: Infrastructure Product innovation Customer relationships	The new Value Proposition targets a large number of historically less profitable, niche Customer Segments – which in aggregate are profitable	The Value Proposition is “giving access” to a company’s existing Customer Segment.	Several Value Propositions are offered to different Customer Segments with different Revenue Streams, one of them being free-of-charge (or nearly free)	Internal R&D is improved with outside partners. Non-monetized internal R&D innovation is transformed into financial value and offered to interested external partners.
**Rationale**	IT and management tools allow different business functions to be optimized separately at lower cost.	Value propositions are tailored for a large number of customers at low cost via IT and operations efficiencies.	An intermediary operating a platform between two or more Customer Segments adds Revenue Streams to the initial model.	Non-paying Customer Segments are subsidized by paying customers in order to attract the maximum number of users.	Acquiring R&D from external sources can be less expensive. Underused innovations have the potential to bring in more revenue.
**Examples**					
Private industry	Banking	Micro-Publishing	Microsoft/Xbox	Red Hat	P&G connect + develop
Health data	HMORN, leveraging healthcare quality improvement IT support infrastructure	ResDAC, providing national operational efficiencies for accessing state Medicaid data	HealthCore, giving access to Anthem data through joint research projects	DARTNet Institute, offering a practice-based research network subsidized by clinical practices paying for quality performance reporting	PCORNet, acquiring high-impact research contracts from external partners to leverage the intrinsic value of the clinical and health plan data networks
**Application for SAFTINet sustainability**	Outsource data infrastructure management Focus academic technical experts on platform innovation (revenue = grants) Hire non-faculty for customer service support	Prioritize access to lower revenue researchers to increase user base (proof-of-concept and publications) De-prioritize targeting single large federally-funded infrastructure grants	Give data access (via contracted reporting like Drug Utilization Reviews) to policy-makers wanting safety-net policy and program evaluations	Clinic partner reporting is highly subsidized (nearly free) by research revenue to attract maximum clinic partners/users (and grow network participation)	Become integrated into national data network consortium for shared innovation (for example, PCORNet) License the ROSITA technology with outside partners to bring in revenue.

The SAFTINet experience extends the set of lessons learned in seeking long-term sustainability and viability of electronic health data research networks. SAFTINet’s effort to apply a business model framework for achieving sustainability is similar to how the DARTNet Institute navigated from an AHRQ-funded research program to a non-profit 501(c) organization for informing practice and improving care [3]. The SAFTINet learning experience is also consistent with key ingredients for successfully building and sustaining data-sharing partnerships as described by Wiehe and colleagues; engagement requires taking a customer-centric, solutions-based approach that involves cyclical, iterative discovery processes [33].

Sustainability planning is also an area of active focus for PCORNet Clinical Data Research Networks (CDRNs). The PCORNet’s task force on health systems interactions and sustainability has a specific aim to “develop models for long-term sustainability of CDRNs in their delivery systems” [34,35]. As we learned in our SAFTINet experience, providing multipurpose resources and demonstrating value to multiple stakeholder types is key. The CDRNs, like SAFTINet, require local data sources to transform their data into a common data model to ensure standardization across the network. Such transformation is a resource-intensive activity that places a significant burden on the health care systems preparing the data. Furthermore, as noted by our stakeholders, providing assurances of data quality is an important factor in long-term use and sustainability of CDRNs such as PCORNet and SAFTINet [36]. The efficiencies offered through support for data harmonization and data quality assurances are a core value of CDRNs. Additionally, demonstrating the ability of CDRNs to support not only research for multiple funders, but for improvement of public health, provides multiple revenue streams for network sustainability [37]. For instance, in May 2018, PCORNet announced a new funding approach aimed at meeting the needs of other research funders such as patient foundations and health care systems as a step toward supporting the sustainability of PCORNet by meeting the needs of multiple funders [38].

The PCORNet network members announced the establishment of an independent non-profit entity, the People-Center Research Foundation to sustain PCORNet’s original mission of patient-centered research, and extending it to explicitly considering the desired outcomes and decision-making perspectives of many stakeholder types, including health system leaders. Details are forthcoming at the time of this submission, but the Foundation is developing a business model that relies on cost reimbursement for access to network members’ data (limited data requests, observational research) and patients (clinical research trials).

## Limitations

A limitation of this case study is that it reflects the experience of a single electronic health data research team and network. Competing data alternatives vary by patient population; as a result, alternative value propositions may resonate more, or less, with different data users and partners within different contextual settings. Instead, we propose a repeatable commercialization-readiness and customer discovery process that can be applied to any data network.

Our experience also underscores the need for on-going customer discovery and product-market fit assessment. New electronic health data platforms and tools are rapidly emerging in a highly dynamic ‘big data’ environment involving major private and public organizations competing for their own sustainability and value as forces of health care transformation. Our learning is relevant to the time period when it was conducted. Since we initiated our customer discovery, there have been numerous data network mergers, new product-service offerings, and new strategic partnerships. Thus, it is critical that environmental scanning be continuous. A challenge federally funded networks face is that there is a focus on demonstrating technical innovation and readiness, often at the expense of demonstrating sustainability-readiness necessary for demonstrating value to those who will ultimately pay to sustain the network. What made this AHRQ-funded project unique, was its emphasis on value optimization and sustainability and AHRQ’s willingness to fund that discovery activity.

## Recommendations For Dialogue

The future promises rapid changes in the national informatics landscape and how researchers, clinicians, patients, policymakers, and other customers will derive value from using electronic health data to transform health care. There is a strong public good argument supporting the appropriateness of ongoing government support for these types of collaborative data network efforts. For example, this is the argument made for financially sustaining large national data networks like the FDA Sentinel Initiative, FDA’s national electronic system for monitoring the safety of FDA-regulated medical products [39]. However, this argument has been more difficult to make to government funders for smaller, regional data networks.

The SAFTINet experience is a case illustration that customer discovery and product-market fit assessment are essential elements for research teams seeking to sustain electronic health data networks and data capabilities when start-up grant funding ends. However, academic scientists are not commonly taught these skills. We suggest two practical ways in which the informatics and data science research community can increase its capabilities for sustaining the value of the networks and health data tools it creates.

1. **Actively develop and foster a data science and clinical and translational workforce knowledgeable in identifying and creating customer value.** This requires a customer-centered business orientation aimed at demonstrating value to decision makers who will ultimately pay for the data infrastructure and products. Recognizing a business-model and commercial-readiness skill gap among academic scientists, the Innovation Corps (I-Corps™) program (www.nsf.gov/i-corps) was launched in 2011 by the National Science Foundation. I-Corps provides immersive customer-discovery and business-model development training for scientists and engineers at academic research centers using the Lean Launchpad methodology developed by Steve Blank [40,41] and the business model canvas popularized by Osterwalder and colleagues [9,32]. Over a thousand teams have participated in the national I-Corps program across multiple federal agencies [42]. I-Corps training is also available to health researchers through the National Institutes of Health Federal Small Business Innovation Research (SBIR)/Small Business Technology Transfer (STTR) and Clinical and Translational Science Awards (CTSA) programs [43]. The National Cancer Institute has developed a similar program called SPRINT.
2. **Fund and require customer discovery and value proposition design as part of electronic health data research and demonstration grants.** Electronic health data research should be incentivized to incorporate customer discovery processes earlier in their network development. For example, based on learning from the SAFTINet experience, we are incorporating customer discovery using the I-Corps program to advance dissemination and product improvement activities for the Accrual-to-Clinical-Trials (ACT) Network [44]. The ACT network is a federated electronic health data network of sites from the National Clinical and Translational Science Award (CTSA) Consortium that was funded by the NIH National Center for Advancing Translational Science (NCATS) to improve cohort discovery and increase participant accrual in clinical trials [44]. To ensure sustainability of the ACT Network, we must demonstrate value to multiple customers – the CTSA academic hubs, end users and health systems who contribute the electronic health data; strategic focus will be critical for success as each of these customers have increasing numbers of competitive alternatives to consider.
  Incorporating customer-centered commercial-readiness frameworks into research proposals is similar to adopting design thinking earlier in the product development pipeline. When PCORI adopted patient-centeredness as a design-thinking principle and required researchers to incorporate patient engagement into their research proposals and dissemination planning, the research community responded. This stimulated greater multi-disciplinary research and fostered innovation in patient engagement strategies and dissemination and implementation science. A similar approach could be taken for stimulating more focus on delivering direct customer value from publicly-funded electronic health data networks and data capabilities.
